# The Emergence and Epidemiology of Haff Disease in China

**DOI:** 10.3390/toxins8120359

**Published:** 2016-12-01

**Authors:** Thomas Y. K. Chan

**Affiliations:** 1 Division of Clinical Pharmacology and Drug and Poisons Information Bureau, Department of Medicine and Therapeutics, Faculty of Medicine, The Chinese University of Hong Kong, Prince of Wales Hospital, Shatin, New Territories, Hong Kong, China; tykchan@cuhk.edu.hk; Tel.: +852-2632-3907; 2 Centre for Food and Drug Safety, Faculty of Medicine, The Chinese University of Hong Kong, Hong Kong, China; 3 Prince of Wales Hospital Poison Treatment Centre, Hong Kong, China

**Keywords:** Haff disease, rhabdomyolysis, crayfish, China

## Abstract

Haff disease is a rare syndrome of unexplained myalgia and rhabdomyolysis occurring within 24 h of consumption of certain types of cooked freshwater fish or crustacean. It is caused by a yet unidentified heat-stable toxin. In the present review of published case studies and official press releases, the main objective is to report the emergence and epidemiology of Haff disease in China. Haff disease first occurred in Beijing in 2000 and in Lianzhou and Liannan, Guangdong Province in 2009. Subsequent outbreaks mostly occurred in the Jiangsu Province—Nanjing, Yangzhou, Huai’an, and Yancheng. Isolated outbreaks occurred in other cities since 2010—Shijiazhuang, Yueyang, Shanghai, Wuhu, Baoding, Shenzhen, and Hong Kong (imported cases from Shenzhen). Outbreaks occurred predominately in the summer. Crayfish accounted for almost all the outbreaks. Two large outbreaks occurred in Lianzhou and Liannan in 2009 (*n* = 54) after eating pomfrets and in Nanjing in 2010 (*n* = 42) after eating crayfish. Other reports or outbreaks involved only 1–9 subjects (median 2 subjects). Variability in individual susceptibility and attack rates were noted, with many subjects remaining asymptomatic despite sharing the same seafood meal as the index cases. Adults were predominately involved. Symptoms occurred within 3–20 h of seafood ingestion, including myalgia, weakness, and, less frequently, nausea, vomiting, abdominal pain, and diarrhea. Myalgia and muscle weakness should normally subside within 2–3 days. Serum creatine phosphokinase became normal within 5–6 days. Abnormal renal function was uncommon. Serious complications (renal failure, multi-organ failure, and prolonged myopathy) and death were rare. In any subjects with unexplained myalgia and rhabdomyolysis, seafood consumption should be included in the history. All suspected cases of Haff disease, including milder presentations, should be reported to public health authorities.

## 1. Introduction

Haff disease is a rare syndrome of unexplained myalgia and rhabdomyolysis occurring within 24 h of consumption of certain types of cooked fish or crustacean, such as buffalo fish [1,2], crayfish [3], and carp fish [4]. It is caused by a yet unidentified heat-stable toxin [2]. It was first described in 1924 in Königsberg (now Kaliningrad) on the Baltic coast among people living near the northern part of the Frisches Haff (now Vistula Lagoon), following the consumption of freshwater eel (*Anguilla anguilla*), pike (*Esox* species), and, especially, burbot (*Lota lota*) [5]. Over the following nine years, an estimated 1000 persons were affected in the summer and fall [6]. In Sweden and the former Soviet Union, outbreaks occurred from 1934 to 1984 [2,6]. In the Americas, Haff disease was known to occur in the U.S. (since 1984) [1] and the Brazilian Amazon (2008–2011) [7,8].

**Figure 1:**
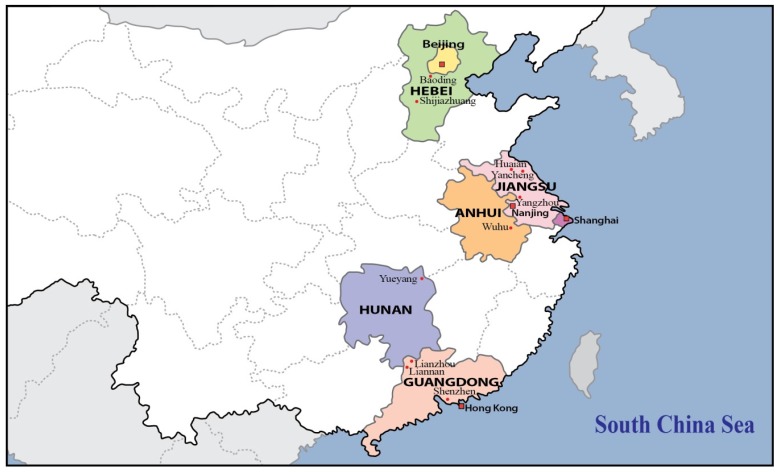
The cities and provinces of China where Haff disease has been reported since 2000.

In China, Haff disease was first reported in Beijing in 2000 [9]. There had since been an obvious increase in the number of reports from other cities. In the present review of published case studies and official press releases, the main objective is to report the emergence and epidemiology of Haff disease in China (Figure 1).

## 2. Literature Search and Case Definition for Haff Disease

To identify relevant articles in indexed medical journals and Chinese medical journals, a search of Medline (2000 to 30 September 2016) and Wanfang Data (2000 to September 2016) was performed, using “Haff disease”, “rhabdomyolysis”, and “crayfish” as search terms. Additional reports from local health authorities were identified from official websites and the medical literature database of our Centre for Food and Drug Safety [10]. Redundant papers substantially reporting the same case series were excluded. Studies providing additional information on the attack rates and possible risk factors were included.

In the published reports, diagnostic criteria based on the history and symptoms might vary. In general, the following case definition for Haff disease was used [1,2]: unexplained myalgia and rhabdomyolysis with a markedly elevated (fivefold or greater increase over normal level) serum creatine phosphokinase (CPK) level and positive cooked seafood consumption history within 24 h. As a result of myoglobinuria, urine can turn red or brown. Myoglobin from skeletal muscle breakdown could cause acute kidney injury. Milder presentations, general and gastrointestinal symptoms also occurred [1,2], sometimes leading to delayed or missed diagnosis.

## 3. Reports of Haff Disease from China

Reports of Haff disease from China during 2000 and 2016 are summarized in Table 1, in relation to the geographical and year of occurrence of the outbreaks. For hospital-based case studies, only the epidemiological data, clinical features, and subject outcomes are presented, since volume repletion and other management for rhabdomyolysis to prevent kidney injury is well known [1,4]. If there were more than one serum CPK result, the peak level was listed. Results of other muscle enzymes are not shown because of their minor importance. Other information useful for characterizing Haff disease, including estimating risks and attack rates, is also presented.

Haff disease first occurred in Beijing in 2000 [9] and then in Lianzhou and Liannan, Guangdong Province, in 2009 [11]. Subsequent outbreaks mostly occurred in the Jiangsu Province—Nanjing in 2010 [12,13,14,15,27,28], 2012 [16], and 2014 [17], Yangzhou in 2012 [18], Huai’an in 2013 [17], and Yancheng in 2015 [19]. Isolated outbreaks occurred in other cities since 2010—Shijiazhuang, Hebei Province, in 2010 [20], Yueyang, Hunan Province, in 2010 [22], Shanghai in 2013 [23], Wuhu, Anhui Province, in 2014 [24], Shenzhen in 2016 [25], and Hong Kong (two imported cases from Shenzhen) in 2016 [26]. In Baoding, Hebei Province [21], the year of occurrence of the outbreaks was not stated in the report of 2014.

Nine reports were from the national [27], provincial [11,16,19], or local [13,17,25,26] food safety [27] and health [11,13,16,17,19,25,26] authorities, including two with additional epidemiology data on the 2010 outbreaks in Nanjing [27,28]. Ten reports were from the local hospitals [9,12,13,14,15,20,21,22,23,24].

As can be seen in Table 1, Haff disease outbreaks occurred predominately in the summer [9,12,13,14,15,16,17,18,22,23,24,25,26]. Two outbreaks had occurred in October [11] and May [19] when the mean daytime temperature in Lianzhou/Liannan and Yancheng was 27 °C and 30 °C, respectively.

Crayfish accounted for almost all the outbreaks of Haff disease in China since 2000 [9,12,13,14,15,16,17,18,19,20,21,23,24,25,26]. Affected subjects generally had ingested 10 pieces or 400 g or more of crayfish. There were two exceptions. Pomfret (*Colossoma brachypomum*) was responsible for the 2009 outbreak in Lianzhou and Liannan [11]. “Lobsters” from Dongting Lake were said to be responsible for the 2010 outbreak in Yueyang [22].

Haff disease outbreaks in two areas should be specially mentioned because of the large number of affected subjects— the October 2009 outbreaks in Lianzhou and Liannan, Guangdong Province (*n* = 54) [11], and the July–September 2010 outbreaks in Nanjing, Jiangsu Province (*n* = 42) [12,13,14,15]. In contrast, all other reports or outbreaks involved only 1–9 subjects (median 2 subjects) (see Table 1). Adults were predominately involved [11,12], likely because crayfish and other seafood are not popular among children and much smaller quantities (if any) were eaten.

Variability in individual susceptibility was also noted since many subjects were asymptomatic despite sharing the same seafood meal as the index cases [15,16,18,19,24,26]. The attack rates among the subjects at different risk levels [11,17,27,28] will be analyzed in detail (see Section 4).

In affected individuals, symptoms occurred within 3–20 h of crayfish ingestion, including myalgia, weakness, and, less frequently, nausea, vomiting, abdominal pain, diarrhea, and other symptoms (see Table 1). However, pomfret ingestion appeared to be associated with a wider range of incubation period (10 min to 41.5 h) and a higher incidence of gastrointestinal features [11]. Myalgia and muscle weakness should normally subside within 2–3 days, and serum CPK became normal within 5–6 days [12]. Abnormal renal function was uncommon (0%–6%) [11,12] because volume repletion and other management for rhabdomyolysis should be effective in preventing kidney injury [1,4].

While the great majority of affected subjects fully recovered, serious complications could occur, including renal failure [14], multi-organ failure [19,23], prolonged myopathy [20], and even death from multi-organ failure [22].

## 4. Discussion

Haff disease, first described in 1924, used to occur along the Baltic coast, Sweden, and the former Soviet Union [2,5,6]. In modern times, this syndrome of unexplained myalgia and rhabdomyolysis occurring within 24 h of consumption of certain types of cooked seafood [1,2,3,4] has remained rare and had not been reported outside the Americas [2,6,7,8]. Isolated cases of milder presentations with less specific symptoms (e.g., weakness, abdominal pain, diarrhea, and vomiting) could be easily missed [21]. It was not known if there had also been under-reporting as in other seafood-borne natural toxin poisoning [10].

The present review describes the emergence and epidemiology of Haff disease in China. As can be seen in Figure 1 and Table 1, both the geographical distribution and the number of reports of Haff disease had increased during 2009–2016 since the first outbreak in Beijing in 2000 [9]. Subsequent outbreaks mostly occurred in the Jiangsu Province [16,17,18,19,28], an eastern coastal province, and recurred in its capital, Nanjing [12,13,14,15,16,17,27,28]. Isolated cases and small outbreaks have occurred in other cities since 2010 [20,21,22,23,24,25,26]. There have so far been two outbreaks with a large number of subjects affected— the October 2009 outbreaks in Lianzhou and Liannan, Guangdong Province (*n* = 54) [11], and July–September 2010 outbreaks in Nanjing, Jiangsu Province (*n* = 42) [12,13,14,15]. Outbreaks occurred in the summer months (Table 1). Crayfish accounted for all outbreaks of Haff disease in China, with the exception of the 2009 outbreaks in Lianzhou and Liannan (pomfret) [11] and the 2010 outbreak in Yueyang (“lobsters” from Dongting Lake) [22]. Adults were predominately involved [11,12].

The clinical presentations of Haff disease were dominated by rhabdomyolysis causing myalgia, weakness, and raised serum CPK (Table 1). Myalgia usually involved could be severe, moderate or mild, involving muscles of the back, shoulder, neck, chest, upper limbs, and lower limbs [27]. Initial presenting symptoms after eating crayfish were very similar between the cases in China and the U.S. [2]. The reason for the higher incidence of gastrointestinal symptoms after pomfret consumption [11] was not known. Milder presentations were common. Almost all affected subjects fully recovered after supportive treatment. However, severe illness with multi-organ failure rarely occurred.

Variations in attack rates and individual susceptibility are often seen in seafood-borne natural toxin poisoning [10]. Largely because isolated or small clusters of cases were previously involved [2], there was a relative lack of such data for Haff disease. Therefore, all the important field studies from China focusing on the attack rates and risk factors for toxicity are presented in detail below.

In Lianzhou, Guangdong Province [11], a retrospective cohort study on 3857 residents revealed that 159 subjects had eaten pomfrets on 26 October 2009, and 50 (excluding the 4 Liannan cases) were ill (an attack rate of 31.5%). In a cluster of 17 male construction workers who had eaten pomfrets together at supper; 15 fell ill (an attack rate of 88.2%). A dose-response relationship was seen between the weight of pomfrets consumed and elevated CPK levels (*r*^2^ = 0.319, *p* = 0.049). Higher weights of fish eaten were associated with higher CPK levels activity and more severe symptoms.

In Nanjing, Jiangsu Province [27], 20 cases (5M, 15F, aged 17–79 years) of Haff disease and 34 persons sharing the crayfish meals were identified during July–August 2010 [27]. Nine persons with muscle pain or raised CPK were excluded. Thus, 20 cases and 25 controls (14M, 11F) were enrolled in this case-control study. The attack rate differed between males and females (26.3% vs. 57.7%, *p* < 0.05). The cases had eaten more crayfish than the controls (20 vs. 5, *p* < 0.001). Eating >10 pieces of crayfish was related to an increased risk of illness (*p* < 0.001).

In Nanjing, Jiangsu Province [28], two adults and one child out of two tables of 4–9 persons were unwell after eating crayfish in a restaurant on 17 August 2010. On-site investigations revealed that >400 kg of crayfish were consumed by >500 people. Two adults out of >500 people were confirmed to have Haff disease (an attack rate of <0.4%).

In Nanjing, Jiangsu Province [17,29], close to 1 million people ate crayfish. With four confirmed cases in 2012 and 2014 and the yearly consumption of several million tons, the risk of Haff disease from eating crayfish among the general public was extremely low.

A heat-stable toxin contained in the seafood is thought to cause Haff disease [2], but attempts to isolate and characterize this toxin have so far failed. Interestingly, mice showed evidence of Haff disease after exposure to implicated fish species. In the October 2009 outbreaks in Lianzhou and Liannan, Guangdong Province [11], mice fed leftover pomfrets showed mental deterioration after 2 h; one out of the 20 mice died after 22 h. In the August 1997 outbreaks in the U.S. [6], mice fed water-soluble extracts from cooked buffalo fish had behavioral changes (consistent with muscle impairment), with red-brown urine in the bladders.

Haff disease must be differentiated from the rhabdomyolysis syndromes caused by palytoxins and other marine toxins with palytoxin-like effects found in certain saltwater fish [30,31,32]. The toxin responsible for Haff disease is found in freshwater crayfish and fish. Haff disease has few, minor neurological symptoms (Table 1). Palytoxins have high acute toxicity with prominent effects on the autonomic and peripheral nervous systems. Unlike Haff disease, the origins (dinoflagellates of the genus *Ostreopsis*) and structures of palytoxins have largely been characterized.

With increases in tourism [26] and global seafood trade and consumption, the geographical distribution and incidence of Haff disease are expected to increase. In subjects with unexplained myalgia and rhabdomyolysis, Haff disease must be included in differential diagnoses, especially if there is a history of recent seafood consumption. Suspected cases, including milder presentation, should be reported to public health authorities for immediate follow-up actions [27,28].

## 5. Conclusions

In China, both the geographical distribution and the number of reports of Haff disease had increased during 2009–2016 since the first outbreak in Beijing in 2000. Subsequent outbreaks mostly occurred in the Jiangsu Province, an eastern coastal province, and recurred in its capital, Nanjing. Isolated cases and small outbreaks occurred in other cities since 2010. Two outbreaks involved a large number of subjects—the October 2009 outbreaks in Lianzhou and Liannan, Guangdong Province (*n* = 54), and the July–September 2010 outbreaks in Nanjing (*n* = 42). Outbreaks occurred in the summer months. Crayfish accounted for most of the outbreaks. Adults were predominately involved.

The clinical presentations of Haff disease were dominated by rhabdomyolysis causing myalgia, weakness, and raised serum CPK. Milder presentations were common. Almost all affected subjects fully recovered. Serious complications (renal failure, multi-organ failure, and prolonged myopathy) and death were rare. Suspected cases should be reported to public health authorities. More research is needed to characterize the responsible toxin, attack rates, and preventive measures.

## Figures and Tables

**Table 1:** Outbreaks of Haff disease after consumption of cooked seafood in China.

City (Reference)	Period	Sex ^a^	Age (Year)	Number of Cases ^e^, Latent Period, Seafood Eaten, Symptoms, Investigations, Progress, *Other Affected Subjects*
Beijing				
[9]	August 2000	2M	(40–42) ^b^	M/40 and M/42 hospitalized; 7 h after eating 500 g of crayfish; moderate or severe myalgia with weakness; serum CPK 2000–10,150 IU/L; symptoms improved 4–72 h later; *1 other boy had mild symptoms*
[9]	August 2000	1F	38	F/38 hospitalized; 12 h after eating 500 g of crayfish; myalgia and weakness; serum CPK 376 IU/L; symptoms improved relapse 8 h after eating left-over crayfish 4 days later; *her child (M/8) had mild myalgia and nausea*
[9]	August 2000	1F	36	F/36 hospitalized; 7 h after eating 500 g of crayfish; severe myalgia, weakness and inability to turn over and talk; symptoms improved 6 h later; *1 other child had mild weakness*
[9]	August 2000	1F	40	F/40 hospitalized; 15 h after eating 400 g of crayfish; moderate myalgia with weakness; serum CPK 6000 U/L; symptoms improved 6 h later
[9]	August 2000	1F	36	F/36 hospitalized; 7 h after eating 20 pieces of crayfish; myalgia and weakness; symptoms improved 12 h later
Lianzhou, Liannan				
[11]	October 2009	36M18F	43 ^c^ (4–74) ^b^	52 adults and 2 children (50 Lianzhou cases, 4 Liannan cases, 44 hospitalized); 10 min–41.5 h (median 7 h) after eating 50–700 g of pomfret (*Colossoma brachypomum*); weakness (83%), myalgia (76%), nausea (63%), abdominal pain (63%), dry mouth (46%), vomiting (46%), dizziness (33%), diarrhea (33%), headache (22%); 85% of 41 cases had abnormal serum CPK (92–11,696, median 1302 U/L); 6% of 36 cases had abnormal serum creatinine; symptom severity/serum CPK related to amounts eaten; home after 1–13 days (median 3.2 days)
Nanjing				
[12]	August 2010	9M14F	35.8 ^d^ (20–79) ^b^	23 subjects hospitalized; 3–12 h after eating crayfish; myalgia (100%), change in urine color (39%), numbness in 4 limbs (17%), nausea (17%), vomiting (13%), abdominal pain (13%), chest tightness (13%), diarrhea (9%), chest pain (9%); peak serum CPK 4655 U/L (mean); normal renal function in all; normal CPK after 5–6 days
[13]	July 2010	1M2F	N.A.	A girl and her mother and father hospitalized; 6–8 h after eating 2, >10 and >20 crayfish; myalgia was more severe in father and mother, with peak serum CPK of 36,360 and 8681 U/L, respectively; all were discharged home within 1 week
[14]	July–August 2010	4M7F	27.6 ^d^ (9–38) ^b^	11 subjects hospitalized; 3–9 h after eating >10 crayfish; myalgia (100%), weakness (82%), change in urine color (64%), nausea (46%), vomiting (18%), and hoarse voice (9%); serum CPK 2013–18,520 U/L (mean 7952 U/L); 1 of the 2 subjects with ↑ serum creatinine required hemoperfusion twice; hospital stay 3–10 days
[15]	July 2010	1M	38	M/38 hospitalized; 6 h after eating crayfish; myalgia and change in urine color; serum CPK 3600 U/L; home after 7 days; *his wife and daughter with similar symptoms after eating crayfish*
[15]	September 2010	1F	21	F/21 hospitalized; 4 h after eating crayfish; myalgia and change in urine color; serum CPK 2176 U/L; home after 16 days; *1 of her 7 colleagues sharing the crayfish meal had similar symptoms*
[16]	August 2012	1M	44	M/44 hospitalized; 5 h after eating 10 crayfish; myalgia, chest discomfort, dyspnea, and thirst; serum CPK 3414 U/L; 4 other subjects sharing the crayfish meal were asymptomatic
[16]	August 2012	1F	31	F/31 hospitalized; 5 h after eating 10 crayfish; myalgia, vomiting, chest discomfort, and dyspnea; peak serum CPK 4992 U/L; *1 other subject who shared the same meal had similar symptoms*
[17]	August 2014	1M1F	32–33	M/33 and F/32; 8–13 h after eating 10–20 crayfish; myalgia; serum CPK 500–3685 U/L
Yangzhou				
[18]	August 2012	1M2F	(30–37) ^b^	1M2F hospitalized; 7–8 h after eating 20–40 crayfish; myalgia; serum CPK 350–5427 U/L; 3 others were well
[18]	August 2012	1M	39	M/39 hospitalized; 7 h after eating 30 crayfish; myalgia; serum CPK 2967 U/L; 4 other subjects were well
Huai’an				
[17]	August 2013	1M	18	M/18; 6 h after eating 30 crayfish; myalgia; serum CPK 622 U/L
Yancheng				
[19]	May 2015	1M	62	M/62 hospitalized; 4–5 h after eating >10 crayfish; nausea, vomiting, abdominal pain, shivering, sweating, chills, and drowsiness; serum CPK 901 U/L, urea 12.4 mmol/L, bilirubin 31.7 μmol/L; ICU care for multi-organ failure before full recovery; 9 other villagers who shared the dinner were asymptomatic
Shijiazhuang				
[20]	2010	1M	26	M/26 hospitalized; after eating crayfish; myalgia and weakness; serum CPK 27,174 U/L, nearly normal renal function; left biceps biopsy revealed rhabdomyolysis; symptoms for >3 months
Baoding				
[21]	N.A.	1F	43	F/43 hospitalized; 20 h after eating crayfish; weakness; peak serum CPK 39,174 U/L; home after 7 days
[21]	N.A.	1M	27	M/27 hospitalized; 15 h after eating a large amount of crayfish; abdominal pain, nausea and vomiting; peak serum CPK 24,356 U/L; home after 10 days
[21]	N.A.	1F	36	F/36 hospitalized; 6 h after eating crayfish; myalgia, weakness; serum CPK 20,110 U/L; home 7 days later
Yueyang				
[22]	July 2010	3M5F	(17–48) ^b^	8 subjects from 3 families hospitalized; 2–3 h after eating “lobsters” from Dongting Lake; dizziness, myalgia, weakness, and chest discomfort; mean serum CPK 457 U/L; symptoms subsided after 3–5 days
Shanghai				
[23]	June 2013	1M	66	M/66 with hypertension hospitalized; 12 h after eating crayfish; myalgia, weakness, limb rigidity, dyspnea, brown-colored urine, and oliguria; serum ALT 4447 U/L, total bilirubin 81.9 μmol/L, creatinine 296 μmol/L, CPK 359 U/L, PT 36.5 s, pH 6.79, PaCO2 10.0 kPa, Hb 9.2 g/dL; CT showed exudates in right middle lobe, lingula and both lower lobes; ICU care for multi-organ failure, he died 2 days later
Wuhu				
[24]	July 2014	1M	41	M/41 hospitalized; 12 h after eating 1 kg of crayfish; myalgia, weakness and brown-colored urine; peak serum CPK 7170 U/L; home 13 days later; other subjects sharing the same meal had no symptoms
[24]	July 2014	1M	43	M/43 hospitalized; 11 h after eating 0.5 kg of crayfish; myalgia and weakness; peak serum CPK 2031 U/L, home 7 days later; other subjects sharing the crayfish meal had no symptoms
Shenzhen				
[25]	August 2016	4MF	N.A.	4 subjects hospitalized; after eating crayfish
Hong Kong				
[26]	August 2016	1F	55	F/55 hospitalized in Hong Kong for 1 day; 4 h after eating crayfish in Shenzhen; myalgia
[26]	September 2016	1F	30	F/30 hospitalized in Hong Kong for 3 days; 5 h after eating crayfish in Shenzhen; myalgia; 2 others were well

^a^ Subjects with marked symptoms; ^b^ ranges, ^c^ median, ^d^ mean; ^e^ Subjects with marked and mild symptoms; CPK reference range of our laboratory 42–186 U/L. N.A. = not available.

## Abbreviations

The following abbreviations are used in this manuscript:

CPK	creatine phosphokinase
